# Notes of the Week

**Published:** 1921-05-21

**Authors:** 


### NOTES OF THE WEEK.

### COLLECTIVE PROPAGANDA.

It is evident from the ready response that has greeted our proposal for a Hospital Pageant that the question of collective propaganda is gaining ground in the minds of those responsible for the administration and management of our voluntary hospitals. Although we do not claim it to be a panacea for all the difficulties which face these institutions to-day, still we believe it is a method open to hospitals to secure increased public support. As we have pointed out in recent issues, there is considerable lack of comprehension on the part of the public concerning the work of the hospitals, and it is only by continued and consistent educative propaganda that interest and enthusiasm can be created. It seems to us that such education rightly belongs to such a body as the British Hospitals Association, which is already doing influential work; its usefulness might be considerably strengthened were it to launch out in the direction of propaganda, and we appreciate that this can be possible only by increased revenue, which might be secured by a larger and more adequate membership subscription on the basis of so much per hospital bed. This fair and equitable arrangement would not place the smaller institutions at a disadvantage. The present methods of isolated appeals are wasteful in time, money and brains, and there is no doubt collective propaganda would help to establish the hospitals on a firm financial basis. With this object always in view The Hospital has for more than thirty years done pioneer work, and will be only too pleased to assist in any movement that will relieve the present unsatisfactory and critical situation. As we go to press we learn that a meeting of hospital secretaries and others is being held on Friday afternoon, May 20, at 3.30 p.m., at these offices, to discuss the possibilities of the Hospital Pageant.

### THE ROYAL FAMILY AND THE HOSPITALS.

The King and Queen and their children have played a larger part in relieving the difficulties of hospitals than, probably, any people unconnected with these institutions can appreciate, for wherever they go the interest of the public follows and the institutions benefit. This must be felt by those responsible for the management of the Hospital for Sick Children and the Victoria Hospital for Children, visited during the last few weeks by their Majesties. The Prince of Wales, the Duke of York and Prince Henry have also recently helped in this way, and the latest Royal visit was that of the Queen to the Royal Free Hospital to inaugurate the new unit of obstetrics and gynæcology, which marks an important stage in the education of medical women. Princess Christian, the President of the Royal Free, another staunch friend of hospitals, received the Queen and read an address. A further address was read by Miss Aldrich Blake, dean of the school, which referred to the untiring efforts of Lady Barrett, M.D., for obtaining the recognition now accorded by the Ministry of Health to the work of women doctors for their own sex and for childhood. It will be remembered that the new unit, which includes sixty-eight beds, is directed by Professor Louise McIlroy, assisted by two full-time medical women.

### DISPENSARY TUBERCULOSIS TREATMENT AND THE RATES.

Metropolitan Borough Councils are not only opposed to the new proposal of the Ministry of Health with regard to the local treatment of tuberculosis, but refuse to put it into operation because of the additional burden upon the local rates. It is maintained that the treatment of tuberculosis is a national matter, and should be borne by the Imperial Exchequer and not by the local authority, as Dr. Addison proposed in his memorandum, which suggests *inter alia* that the borough councils shall provide for any extra nourishment required by the patient; such expenditure not to exceed £2 per head of the population of each borough per annum. At Islington this is estimated at £3,460 a year, and in other poor boroughs this will be exceeded. The local authorities urge that during the past few years the additional burdens cast upon them have grown to an alarming degree, and that if there is to be any reduction in rates the authorities must refuse to carry them out. The first thing, therefore, the councils have decided to do is to resist to the utmost this additional burden cast upon them by the Ministry of Health, leaving the Government to carry out and pay for their own new schemes.

### PICTURES HELP HOSPITAL APPEAL.

Good progress is being made with the building additions to the Royal Waterloo Hospital, but funds are still urgently needed. The total cost will, it is estimated, amount to £80,000. When completed the hospital will have the advantage of a larger out-patients' department, more beds for in-patients, and a Home for nurses. This latter is particularly needed, the present accommodation being uncomfortable and inadequate. The Special Appeal Committee, constituted under the chairmanship of the Earl of Athlone, has worked hard, and the sum now required to complete the appeal is £59,000. The three striking and artistic cartoons, which occupy a prominent position on the exterior of the hospital in Waterloo Bridge Road, are the work of Miss Laura Knight, and were designed by the artist in compliance with a special request of Princess Alice, Countess of Athlone. The Secretary informs us that the silent appeal the pictures make has stimulated the generosity of the public, the contents of the boxes outside the hospital having shown a gratifying increase since they were installed.

### NEW SECRETARY OF THE GUEST HOSPITAL.

The temporary arrangement whereby Mr. Hurst, formerly assistant, has been acting as secretary to the Guest Hospital, Dudley, in consequence of Mr. Bird's resignation, has now terminated. The new secretary is Mr. James Gough, a chartered accountant, and already a member of the Finance Committee. He has, indeed, been honorary auditor to the hospital for some time, and the Board has agreed to his condition that he shall have competent assistance. The appointment was made unanimously.

### BETTER NEWS FROM BOLINGBROKE HOSPITAL.

Thanks to grants from the National Relief Fund and other funds, the Bolingbroke Hospital, Wandsworth Common, is no longer oppressed with an overdraft, and is in a stronger position than it was a year ago. The Balham and Tooting Traders' Association has subscribed £2,375 and the schools of the district have raised over £1,000. These sources of revenue should be permanent, and, if so, are likely to increase. Since 1880, when the work began, the hospital has made considerable progress. In the eighties it was one of those institutions where the resident medical officer was also the honorary secretary. It is perhaps strange that that practice did not develop in the other direction and prove the germ of the medical superintendent.

### AN INTERESTING DINNER.

We would again remind those interested in health matters that the annual dinner of the Federation of Medical and Allied Societies is being held at the Café Royal on Thursday, May 26. The chief guest will be Dr. Addison, M.P., in recognition of the services he has rendered. Sir Alfred Mond will be present, as also will members of the medical group in Parliament. Those wishing to attend should make immediate application to the General Secretary, 5 Vere Street, London, W.

### NEW TUBERCULOSIS HOSPITAL, MONMOUTHSHIRE.

Lord Tredegar, who lately purchased Cefn Mably, the historic house near Castleton, is said by the *Western Mail* to be willing to co-operate with the King Edward VII. Welsh National Memorial Association in converting the house to a sanatorium for Monmouthshire. The war prevented the building of a similar hospital with 100 beds on the site offered by Mr. Hanbury near Pontypool Road Junction. An attempt to carry through the scheme last year was abortive because of the increase in its cost. Then the possibility of Cefn Mably was suggested, and after Lord Tredegar's interest had been gained the estimate for its conversion and extension was £30,000, or one-sixth of the previously rejected scheme. The house is said to have been built in the twelfth century, and Charles the First stayed there before the Battle of Worcester.

### A DEFICIT CLEARED OFF.

As the result of strenuous efforts only £1,161 is needed to complete the appeal of the London Homœopathic Hospital for £16,831 required to clear off the accumulated deficits to the end of 1916. Provided that £661 is raised by July 31, Lord Dysart has kindly promised to give £500. The success of the appeal is thus practically assured.

### ARKLOW COTTAGE HOSPITAL OPENED.

Ireland gains another cottage hospital with the opening of the Countess of Wicklow Memorial Cottage Hospital, Arklow. The Most Rev. Dr. Bernard, Provost of Trinity College, Dublin, performed the opening ceremony. The hospital has been endowed with £5,000 by Lady Wicklow's relatives and friends, and all classes seem to have united in its foundation, which will be of great service to the district. The honorary secretary is the Rev. P. W. Coster.

### THE SHEFFIELD CONTRIBUTION SCHEME.

The Sheffield Rotary Club is an institution at which informative addresses are regularly given upon local public affairs. A recent speaker was Mr. S. R. Lamb, organising secretary of the Sheffield Joint Hospitals Council, who dealt with the indebtedness of the four voluntary hospitals, now amounting to £92,000. The Council, said Mr. Lamb, is asking all sections of the community, especially those which use the hospitals, to contribute regularly. The scheme is for all in works, shops, and offices to give a penny in the pound, and for employers to add not less than one-third of the total as an annual subscription. In acknowledgment, contributors and their dependants, when in hospital, will not be asked to contribute to the cost of their maintenance. Accommodation for paying wards cannot be spared at present. To meet the workpeople's wishes it is hoped that the joint contribution scheme will also secure, in approved cases, institutional treatment on favourable terms at special institutions and convalescent homes. Other speeches were made by Mr. C. H. Wilson and Mr. F. Osborn, members of the Council, and Mr. Lamb's speech at the Rotary Club should help him in his task by interesting a wider public in the difficulty for which the new scheme is the most promising remedy. The building and allied trades are now discussing the adoption of the contribution scheme, and other trade associations will probably fall into line.

### THE IRISH APPEAL.

A correspondent writes: Disappointment is expressed in the Irish Press at the result of the effort to raise £100,000 to pay off hospital war debts. The sum of £35,000 has been collected, and, taking all the circumstances into account, this is very creditable. In the first place, several hospitals held aloof from the movement, preferring to appeal to the public on their own account. In this they probably acted with wisdom, if not with *esprit de corps*, for they have their own *clientèle* on whose generosity they know that they can always count. Secondly, the grand fête was held in October, a most inauspicious month, when the weather was, as might have been anticipated, adverse. Thirdly, and probably of greatest importance, the Committee did not include as active workers a considerable number of those influential Irish people whose efforts, when put forth, naturally turn into money. The wonder is that £35,000 was realised.

